# The Use of Propofol versus Dexmedetomidine for Patients Receiving Drug-Induced Sleep Endoscopy: A Meta-Analysis of Randomized Controlled Trials

**DOI:** 10.3390/jcm10081585

**Published:** 2021-04-09

**Authors:** Yi-Ting Chen, Cheuk-Kwan Sun, Kuan-Yu Wu, Ying-Jen Chang, Min-Hsien Chiang, I-Wen Chen, Shu-Wei Liao, Kuo-Chuan Hung

**Affiliations:** 1 Department of Anesthesiology, Chia-Yi Chang Gung Memorial Hospital, Chia-Yi 61363, Taiwan; rita815221@gmail.com; 2 Department of Emergency Medicine, E-Da Hospital, Kaohsiung 83301, Taiwan; lawrence.c.k.sun@gmail.com; 3 College of Medicine, I-Shou University, Kaohsiung 84001, Taiwan; 4 Department of Urology, National Cheng Kung University Hospital, Tainan 70101, Taiwan; hn85386039@gmail.com; 5 College of Medicine, National Cheng Kung University, Tainan 70101, Taiwan; 6 Department of Anesthesiology, Chi Mei Medical Center, No.901, ChungHwa Road, YungKung Dist, Tainan 71004, Taiwan; 0201day@yahoo.com.tw (Y.-J.C.); mavising@gmail.com (I.-W.C.); buzzer176@gmail.com (S.-W.L.); 7 College of Health Sciences, Chang Jung Christian University, Tainan 71101, Taiwan; 8 Department of Anesthesiology, Kaohsiung Chang Gung Memorial Hospital, Chang Gung University College of Medicine, Kaohsiung 83301, Taiwan; ducky0421@gmail.com; 9 Department of Health and Nutrition, Chia Nan University of Pharmacy and Science, Tainan 71710, Taiwan

**Keywords:** dexmedetomidine, propofol, drug-induced sleep endoscopy, hypoxemia

## Abstract

The sedation outcomes associated with dexmedetomidine compared with those of propofol during drug-induced sleep endoscopy (DISE) remains unclear. Electronic databases (i.e., the Cochrane controlled trials register, Embase, Medline, and Scopus) were searched from inception to 25 December 2020 for randomized controlled trials (RCTs) that evaluated the sedation outcomes with dexmedetomidine or propofol in adult patients diagnosed with obstructive sleep apnea (OSA) receiving DISE. The primary outcome was the difference in minimum oxygen saturation (mSaO2). Five RCTs (270 participants) published between 2015 and 2020 were included for analysis. Compared with dexmedetomidine, propofol was associated with lower levels of mSaO2 (mean difference (MD) = −7.24, 95% confidence interval (CI) −12.04 to −2.44; 230 participants) and satisfaction among endoscopic performers (standardized MD = −2.43, 95% CI −3.61 to −1.26; 128 participants) as well as a higher risk of hypoxemia (relative ratios = 1.82, 95% CI 1.2 to 2.76; 82 participants). However, propofol provided a shorter time to fall asleep and a lower risk of failed sedation compared with dexmedetomidine. No significant difference was found in other outcomes. Compared with propofol, dexmedetomidine exhibited fewer adverse effects on respiratory function and provided a higher level of satisfaction among endoscopic performers but was associated with an elevated risk of failed sedation.

## 1. Introduction

Obstructive sleep apnea (OSA), which is characterized by repeated complete or partial upper airway collapse, is an increasingly common sleep disorder, leading to cyclical hypoxemia and disrupted sleep. A previous study has demonstrated that patients with OSA had certain anatomical anomalies including a longer mandibular plane–hyoid distance, a smaller posterior airway space area, and a larger tongue volume compared with those in individuals without OSA [1]. Another study has attributed the blockade of air flow to a high collapsibility of the upper airway (i.e., a high passive critical closing pressure, Pcrit) [2]. Treatments of choice for OSA include continuous positive airway pressure (CPAP) [3], weight loss, oral appliances (e.g., mandibular advancement devices) [4,5], and surgical interventions [6,7].

For patients refractory to or with poor tolerance to conservative treatments (e.g., CPAP), surgical procedures may be indicated for those with obvious anatomical obstructions [8]. Accurate upper airway evaluation (e.g., site and pattern of airway obstruction) is crucial to surgical decision-making and successful outcomes [9,10,11]. Drug-induced sleep endoscopy (DISE), which was introduced in 1991 [10], is considered to be a safe and practical technique to evaluate the location of dynamic upper airway collapse during sleep [12]. It is useful for preoperative planning to optimize treatment option selection or guide procedure modifications for patients with OSA [13]. Previous studies have investigated the use of several anesthetics such as propofol [14], midazolam [15], and dexmedetomidine [16] as sedating agents for the DISE procedure to identify the site of airway collapse. Nevertheless, all agents are associated with specific advantages and disadvantages. A previous systematic review indicated that dexmedetomidine seemed to offer a more stable cardiopulmonary profile for the procedure, while propofol not only had a quicker onset and a shorter half-life but also enabled the detection of a larger degree of obstruction [17]. However, the findings of that review were mostly based on case series (i.e., eight out of the ten included studies) with the inclusion of only one randomized controlled trial (RCT) and one prospective cohort study. On the other hand, a meta-analysis was not performed for comparing the clinical merits and downsides in this clinical setting. Accordingly, the current study aimed at objectively comparing the efficacy of sedation induction and safety profile (e.g., oxygen desaturation, hypotension) as well as the subjective level of satisfaction among the endoscopic performers (i.e., surgeons) and patients when different anesthetic agents were used for the DISE procedure.

## 2. Materials and Methods

The present meta-analysis was conducted in accordance with the Preferred Reporting Items Systematic Reviews and Meta-Analysis (PRISMA) guidelines [18] and was registered with PROSPERO (CRD42020203715).

### 2.1. Search Strategy

The databases of the Cochrane Central Register of Controlled Trials (CENTRAL), Medline, Embase, and Scopus were searched for RCTs using the following keywords: “DISE”, “sleep endoscopy”, “drug-induced sleep endoscopy”, “Propofol”, “Diprivan”, “2, 6-diisopropylphenol”, “Dexmedetomidine”, “precedex”, “Dexdor”, and “RCT” from inception to 25 December 2020. The reference lists in all the retrieved articles were manually searched to screen for other studies not found during our electronic screening. No publication date was applied, but only trials published in English were included. The search strategies and syntax for one of these databases (i.e., Embase) can be found in Supplemental Table S1.

### 2.2. Study Selection Criteria and Data Extraction

Two reviewers (YTC, CKS) independently examined the titles and abstracts of the articles to identify potentially eligible studies comparing the sedative outcomes with the use of propofol or dexmedetomidine in patients receiving DISE. The criteria for eligibility of RCTs included (1) adult patients (age ≥ 18 years) undergoing DISE; (2) propofol- or dexmedetomidine-based agents were given to induce sedation during DISE. There were no restrictions on dose or anesthetic technique (e.g., bolus or infusion technique). The exclusion criteria were (1) studies on the pediatric population, (2) those in which information regarding sedative outcomes was unavailable, and (3) studies which use propofol and dexmedetomidine as a combination for DISE. Two authors (KYW, YJC) independently read the full text of the trials to assess their eligibility for the final analysis. Two reviewers (MHC, IWC) independently performed the extraction of data that included year of publication, primary author, sample size, study setting, patient characteristics, anesthetic technique, and sedative outcomes (e.g., episode of desaturation). In the situation of disagreements, a third author (KCH) was involved until a consensus was reached. The corresponding authors of the included studies that did not provide data on primary or secondary outcomes were contacted for further information.

### 2.3. Primary Outcome, Secondary Outcomes, and Definitions

The primary outcome was the minimum oxygen saturation (mSaO2) during sedation, while the secondary outcomes included the risks of oxygen desaturation and failed sedation procedure, time to achieve sufficient sedation, duration of endoscopic examination, episodes of hypotension or bradycardia, as well as the satisfaction of patients or endoscopic performers. An episode of oxygen desaturation was defined as one with an arterial oxygen saturation (SaO2) < 90%. An episode of hypotension or bradycardia was defined as criteria of each trial (Supplemental Table S2).

### 2.4. Assessment of Risk of Bias for Included Studies

Two authors (SWL, KCH) evaluated the risk of bias for the included trials using the criteria described in the Cochrane Handbook for Systematic Reviews of Interventions [19]. Disagreements were solved through discussion. After analyzing the overall risk of bias of all the included studies and the risk of bias of individual studies, we rated the potential risk of bias by assigning a score of “low”, “high,” or “unclear” to each trial.

### 2.5. Statistical Analysis

A random effects model was adopted to compute the risk ratios (RRs) together with the 95% confidence intervals (CIs) for dichotomous outcomes. For pooling dichotomous data and calculating the pooled RRs with 95% CIs, the Mantel–Haenszel (MH) method was used. For continuous outcomes, the selected effect size was the standardized mean difference (SMD). The I^2^ statistic was utilized for the assessment heterogeneity (low: 0% to 50%; moderate: 51% to 75%, high: 75% to 100%). When three or more studies reported on a particular outcome, sensitivity analyses were performed by omitting the studies from the meta-analysis one at a time to explore the potential impact of a single trial on the overall results. When 10 or more studies reported on a particular outcome, the funnel plots were inspected to investigate the probabilities of reporting and publication bias. A probability value less than 0.05 was considered statistically significant for all analyses. The Cochrane Review Manager (RevMan 5.4; Copenhagen: The Nordic Cochrane Centre, The Cochrane Collaboration, 2014) was applied for data synthesis.

## 3. Results

### 3.1. Study Selection

**Figure 1:**
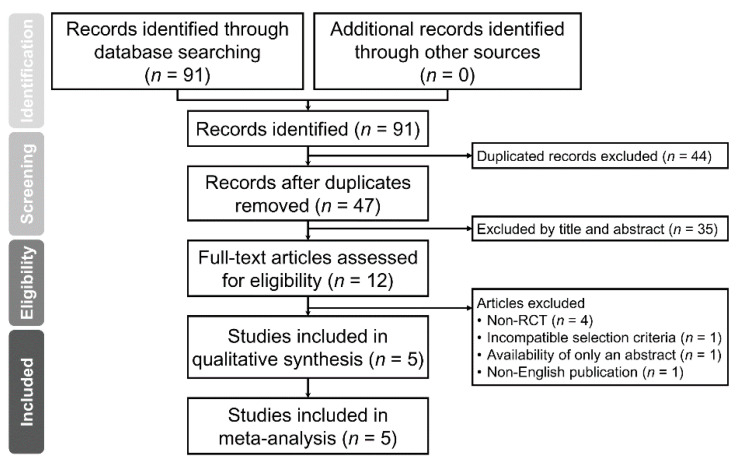
Meta-analysis flowchart for selecting eligible studies. RCT: randomized controlled trial.

The Preferred Reporting Items for Systematic reviews and Meta-Analyses (PRISMA) flow diagram summarizing the reasons for study exclusion is shown in Figure 1. Of a total of 91 eligible records retrieved from our database search, 44 were deemed ineligible because of being duplicates. Thirty-five records were then excluded after initial screening of the titles and abstracts. Overall, 12 reports were considered to be relevant. After full-text reading, another 7 articles were excluded due to non-RCT designs (*n* = 4), incompatible selection criteria (*n* = 1), availability of abstract only (*n* = 1), and non-English publication (*n* = 1). Finally, 5 randomized trials [20,21,22,23,24] in total were included in the present meta-analysis (Figure 1).

### 3.2. Characteristics of Included Studies

Five RCTs involving 270 participants published between 2015 and 2020 were analyzed. Characteristics of the studies are detailed in Table 1. The number of participants ranged from 40 to 88. Four studies compared the sedative outcomes in patients receiving propofol or dexmedetomidine [20,21,22,23], while one trial investigated these outcomes in patients being given propofol/remifentanil or dexmedetomidine/remifentanil combination [24]. The goal of sedation varied among the five studies (Table 1), and the depth of anesthesia was monitored with different approaches, namely, bispectral index in three trials [21,22,24], Ramsey Sedation Scores (RSS) ≥ 3 in one study [23], and disappearance of the eyelash reflex in the other trial [20]. The sedation technique included target-controlled infusion technique [24], infusion technique [21,22,23], and a combination of bolus and infusion technique [20].

### 3.3. Risk of Bias Assessment

**Figure 2:**
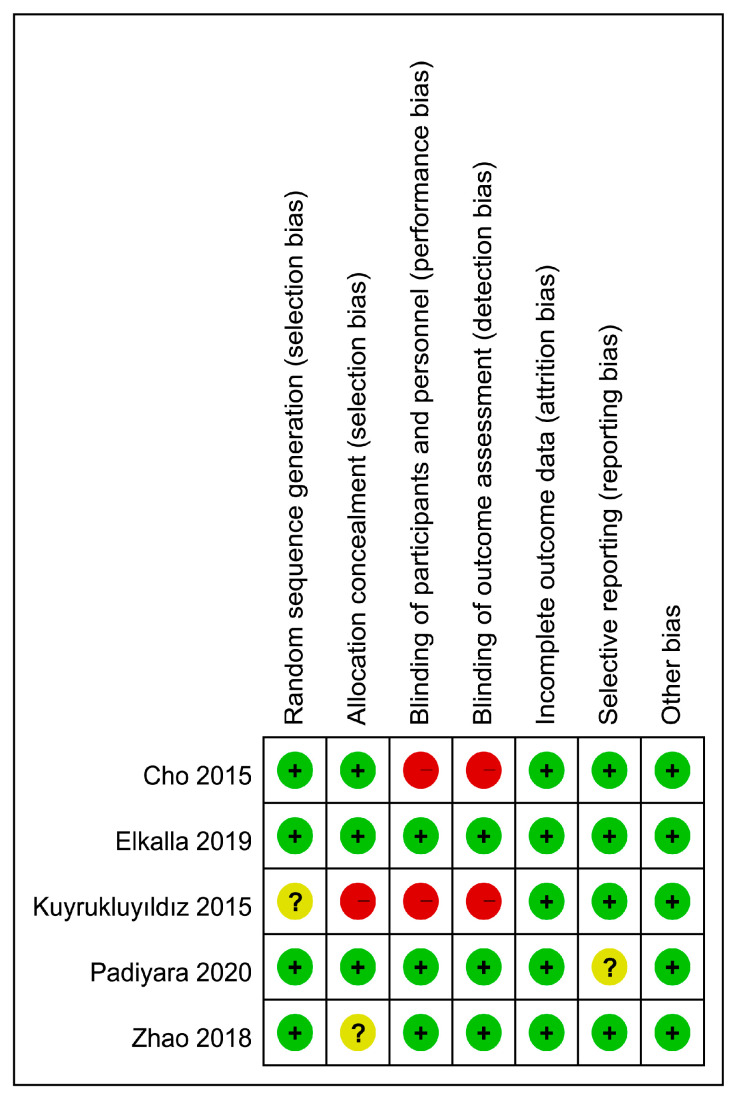
Risks of bias of individual studies.

**Figure 3:**
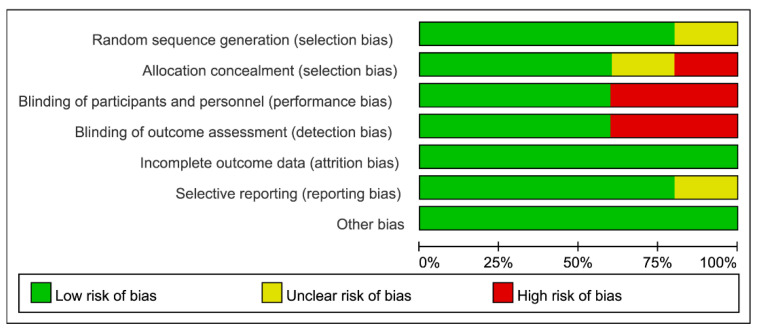
Overall risks of bias of the five included studies.

The risks of bias of individual trials and the overall risk of bias are depicted in Figure 2 and Figure 3, respectively. Regarding the risk of random sequence generation, one trial did not mention this information specifically [21]. For allocation concealment, the risk of bias in two studies was considered to be uncertain or high because of no specific statement addressing this issue [20,21]. Two trials were regarded as carrying the risk of performance bias and detection bias as they did not provide irrelevant information [21,24]. One trial was suggested to carry the risk of reporting bias because the registered information was unavailable, and only some information regarding primary and secondary outcomes was available [22]. Detailed information about bias evaluation of the included studies is provided in Supplemental Table S3.

### 3.4. Sedative Outcomes

#### 3.4.1. Difference in Minimum Oxygen Saturation and Risk of Desaturation

**Figure 4:**
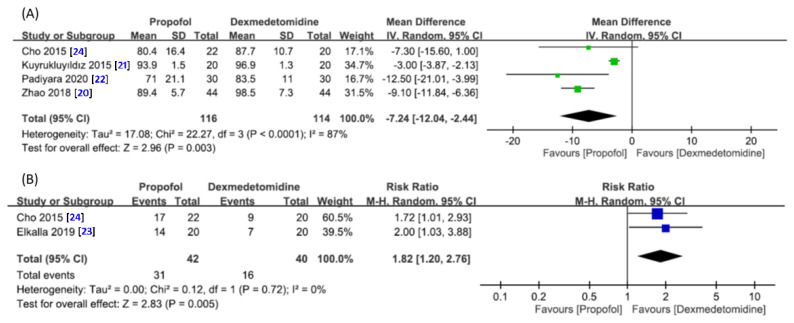
Forest plot for comparing (**A**) the minimum oxygen saturation and (**B**) risk of oxygen desaturation during drug-induced sleep endoscopy between propofol and dexmedetomidine groups. CI, confidence interval; IV, inverse variance; M–H, Mantel–Haenszel.

Four studies involving a total of 230 patients (propofol group, *n* = 116 vs. dexmedetomidine group, *n* = 114) were available for the analysis [20,21,22,24]. A forest plot demonstrated a lower mean mSaO2 during DISE in the propofol group compared with that in the dexmedetomidine group (MD = −7.24, 95% CI −12.04 to −2.44, *p* = 0.003; I^2^ = 87%) (Figure 4A). Sensitivity analysis did not show a significant impact on outcome by omitting certain trials.

Two studies with 82 patients in total (propofol group, *n* = 42 vs. dexmedetomidine group, *n* = 40) provided relevant data for analyzing the risk of oxygen desaturation (Figure 4B) [23,24]. Pooled analysis showed a higher risk of oxygen desaturation with the use of propofol compared with dexmedetomidine (RR = 1.82, 95% CI 1.2 to 2.76, *p* = 0.005; I^2^ = 0%) (Figure 4B).

#### 3.4.2. Risk of Failed Sedation Procedure for Drug-Induced Sleep Endoscopy

**Figure 5:**
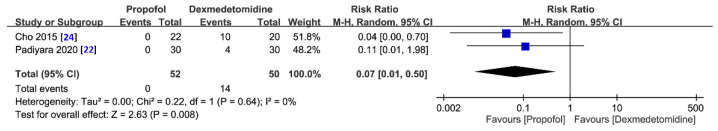
Forest plot for comparing the risk of failed sedation for drug-induced sleep endoscopy between propofol and dexmedetomidine groups. CI, confidence interval; M–H, Mantel–Haenszel.

The forest plot on two available studies involving a total of 102 patients (propofol group, *n* = 52 vs. dexmedetomidine group, *n* = 50) [22,24] is shown in Figure 5, which demonstrated a lower risk of failed sedation in the propofol group compared with that in the dexmedetomidine group (RR = 0.07, 95% CI 0.01 to 0.50, *p* = 0.008; I^2^ = 0%) (Figure 5).

#### 3.4.3. Time to Fall Asleep and Duration of Endoscopic Examination

**Figure 6:**
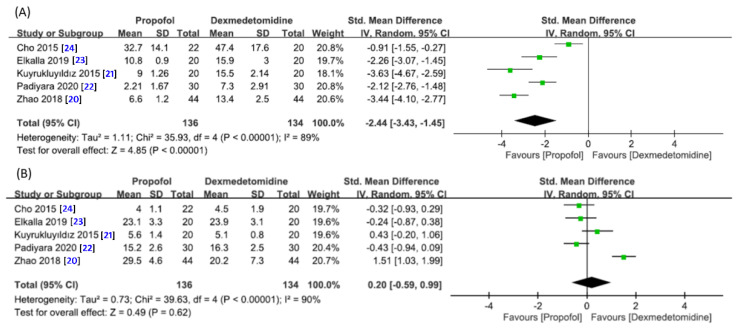
Forest plot for the comparison of (**A**) time to fall asleep and (**B**) duration of endoscopic examination between propofol and dexmedetomidine groups. CI, confidence interval; IV, inverse variance; Std, standardized.

Five studies with 270 patients in total (propofol group, *n* = 136 vs. dexmedetomidine group, *n* = 134) contained data for the analysis [20,21,22,23,24]. A forest plot is presented in Figure 6A, which revealed a faster drug-induced sedation in the propofol group than that in the dexmedetomidine group (SMD = −2.44, 95% CI −3.43 to −1.45, *p* < 0.00001; I^2^ = 89%). No significant impact on outcome was noted by omitting certain trials on sensitivity analysis.

The duration of endoscopic examination is shown in Figure 6B. The forest plot demonstrated no significant difference in time required for DISE between the two groups (SMD = 0.20, 95% CI −0.59 to 0.99, *p* = 0.62; I^2^ = 90%) (Figure 6B). Sensitivity analysis showed no significant influence on outcome by omitting certain trials.

#### 3.4.4. Risk of Hypotension or Bradycardia

**Figure 7:**
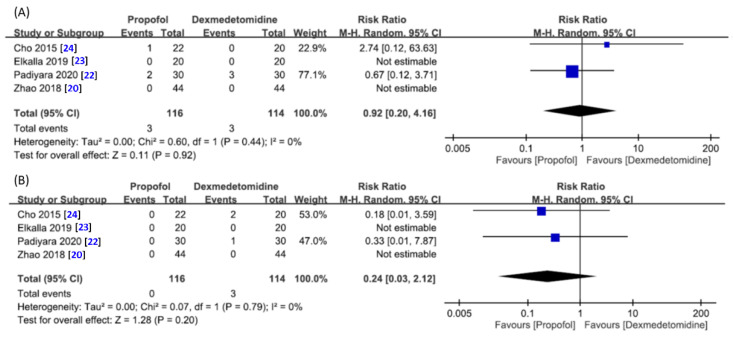
Forest plot for the comparison of (**A**) risk of hypotension or (**B**) risk of bradycardia during drug-induced sedation endoscopy between propofol and dexmedetomidine groups. CI, confidence interval; M–H, Mantel–Haenszel.

Four studies with a total of 230 patients (propofol group, *n* = 116 vs. dexmedetomidine group, *n* = 114) offered relevant data for the analysis of these hemodynamic outcomes [20,22,23,24]. The forest plot demonstrated a comparable risk of hypotension (RR = 0.92, 95% CI 0.20 to 4.16, *p* = 0.92; I^2^ = 0%) (Figure 7A) or bradycardia (RR = 0.24, 95% CI 0.03 to 2.12, *p* = 0.20; I^2^ = 0%) (Figure 7B) between the two groups.

#### 3.4.5. Satisfaction of Endoscopic Performers or Patients with the Sedation Technique

**Figure 8:**
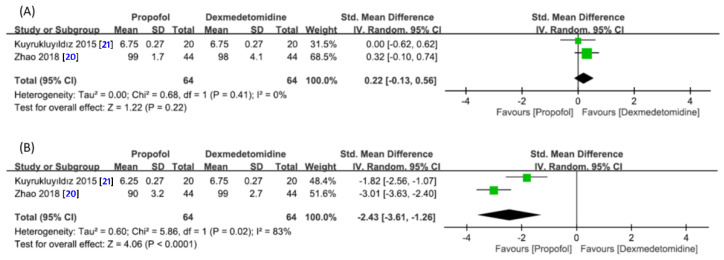
Forest plot for the comparison of satisfaction of (**A**) patients and (**B**) endoscopic performers between propofol and dexmedetomidine groups after drug-induced sedation. CI, confidence interval; IV, inverse variance; Std, standardized.

Two studies involving a total of 128 patients (Propofol group, *n* = 64 vs. dexmedetomidine group, *n* = 64) [20,21] were available for analysis. The forest plot demonstrated that the levels of satisfaction among patients receiving the sedation technique were comparable between the two agents (SMD = 0.22, 95% CI −0.13 to 0.56, *p* = 0.22; I^2^ = 0%) (Figure 8A), while the endoscopic performers were less satisfied with the propofol compared with dexmedetomidine (SMD = −2.43, 95% CI −3.61 to −1.26, *p* < 0.0001; I^2^ = 83%) (Figure 8B).

## 4. Discussion

The current meta-analysis compared the objective and subjective outcomes of using propofol and dexmedetomidine for sedation induction during the DISE procedure because both agents have been reported to offer the advantages of a relatively short half-life and easy titration [25].The present study on five RCTs showed that the minimum oxygen saturation and the satisfaction among endoscopic performers was higher, while the risk of oxygen desaturation was lower in patients with OSA receiving dexmedetomidine as the hypnotic agent compared with the corresponding parameters when propofol was used. Our results also showed that the use of dexmedetomidine carried the risks of failed sedation and delayed onset for sedation despite its fewer respiratory depressive effects. On the other hand, we demonstrated no significant differences in the duration of sedation endoscopy, hemodynamic profiles, and patient satisfaction between the propofol and dexmedetomidine groups.

Through sleep induction, DISE enables preoperative exploration of the obstruction sites (e.g., tongue base and/or epiglottis, tonsils, lateral pharyngeal wall, soft palate) which is pivotal to surgical success for OSA [26]. Midazolam and propofol are both hypnotic agents widely used for sleep endoscopy, while dexmedetomidine is less frequently used [27]. Dexmedetomidine exerts its hypnotic effect on the locus coeruleus through activating its central pre- and postsynaptic α2-receptors, thereby eliciting a state of unconsciousness similar to that during natural sleep [28]. Additionally, dexmedetomidine has the merits of being anxiolytic, analgesic-sparing, and sympatholytic with minimal depression of respiratory function [28]. As the applications of dexmedetomidine have increased for DISE, several RCTs have attempted to compare the benefits and disadvantages between propofol and dexmedetomidine [20,21,22,23,24]. Although a previous systematic review, which mostly focused on RCTs and retrospective studies, supported the use of dexmedetomidine for DISE because of its overall safer and more stable cardiopulmonary profile [17], our meta-analysis is the first to analyze the cardiopulmonary status, risk of failed sedation, procedure time, and satisfaction of patients and endoscopic performers based on RCTs.

Although reproducing a state of sleep for diagnosis of OSA unavoidably increases the risk of oxygen desaturation [17], a previous report focusing on healthy male volunteers showed that dexmedetomidine-induced sedation could suppress ventilatory responses to hypoxia and hypercapnia to an extent similar to that of propofol [29]. On the other hand, despite fewer respiratory depressive effects associated with the use of dexmedetomidine for sedation in other studies, episodes of airway obstruction and apnea have been reported [29,30]. Consistently, the current meta-analysis demonstrated that dexmedetomidine-induced sedation was associated with less respiratory suppression as reflected by a higher level of mSaO2 and a lower risk of oxygen desaturation in patients with OSA compared with propofol. The issue of anesthesia-related hypoxemia is critical because not only have episodes of postoperative hypoxemia been reported to increase the risk of cerebral dysfunction, wound infection, and cardiac arrhythmias [31], but hypoxemia has also been found to be a potential risk factor for cardiac ischemia during procedural sedation (e.g., colonoscopy) [32,33]. Indeed, one of the included studies [24] demonstrated that two patients receiving propofol–remifentanil combination during DISE developed a cardiac arrhythmia including atrial premature complex and sinus arrhythmia. Furthermore, taking into account the current evidence that supports a strong association between OSA and cardiovascular comorbidities (e.g., coronary artery disease, arrhythmia, and sudden cardiac death) [34], avoidance of hypoxemia during DISE may be recommended to enhance patient safety.

In the current meta-analysis, two RCTs reported the occurrence of inadequate sedation level with dexmedetomidine [22,24]. Our pooled results showed a lower risk ratio of failed procedural sedation with propofol compared with dexmedetomidine (RR = 0.07, 95% CI 0.01 to 0.50, *p* = 0.008). In concert with this finding, a review of literature revealed sporadic reports on failure of dexmedetomidine to consistently achieve deep sedation in critical care settings [35,36]. Although the sedation goal for DISE may vary among endoscopy performers [27], most reports attempted to obtain a bispectral index (BIS) < 60 [37,38], or even 40 in some studies [39]. Failed or inadequate sedation during DISE may carry the risk of underestimating the degree of upper airway collapse, which may vary with the depth of sedation [40]. Therefore, the current study supports that a combination of dexmedetomidine with other hypnotic agents or the use of BIS as a monitoring approach may be considered optimal for DISE. At the other end of the spectrum, excessive sedation during DISE may increase the risk of overestimating the degree of upper airway collapse. One included trial reported the potential risk of false positivity in the diagnosis of sleep apnea hypopnea syndrome associated with the use of propofol [20]. Large-scaled studies are warranted to address these issues.

In terms of sedation efficacy, the current meta-analysis revealed that the use of propofol was associated with a shorter time to fall asleep (SMD = −2.44, 95% CI −3.43 to −1.45, *p* < 0.00001; I^2^ = 89%). As the use of dexmedetomidine may be related to a delay in the time to fall asleep, assessment of the adequacy of sedation is crucial before the implementation of surgical procedures. In addition, the significantly increased duration of endoscopic examination associated with the use of propofol for DISE [24] may raise a serious clinical concern as sedation-associated hypoxemia may contribute to cardiac morbidities during similar endoscopic procedures (e.g., colonoscopy) [32,33]. Therefore, for procedures that carry a high risk of oxygen desaturation (e.g., DISE), the procedure time should be minimized. The pooled results in current meta-analysis demonstrated no significant difference in procedural time required for DISE in patients receiving propofol or dexmedetomidine (SMD = 0.20, 95% CI −0.59 to 0.99, *p* = 0.62). However, the high heterogeneity in our results on the time to fall asleep and procedural time warrants further large-scale trials for validation of the findings.

A previous meta-analysis has reported a higher level of patient satisfaction related to sedation with propofol compared to dexmedetomidine in those undergoing gastrointestinal endoscopy [41]. However, the current meta-analysis demonstrated comparable levels of satisfaction among patients receiving propofol or dexmedetomidine. Such discrepancy may be attributed to the aggressiveness of the procedure. Compared with DISE, gastrointestinal endoscopy may cause a higher degree of patient discomfort that requires a stronger hypnotic agent for procedure conduction. In this way, a delay in falling asleep with dexmedetomidine may cause patient dissatisfaction. In contrast, we found that endoscopic performers were significantly more satisfied with dexmedetomidine compared with propofol (SMD =−2.43, 95% CI −3.61 to −1.26, *p* < 0.0001). The characteristic sedation status achieved by dexmedetomidine similar to that of natural physiological sleep among conscious patients without notable respiratory depression [28], together with the high risk of oxygen desaturation and the increased probability of airway manipulation associated with propofol use, may contribute to the observed higher satisfaction with dexmedetomidine compared to that with propofol among endoscopic performers.

The current meta-analysis had its limitations that need to be taken into consideration for accurate interpretations of its findings. First, the number of included studies was too small to assess the potential publication bias. Second, differences in sedation technique (e.g., infusion or bolus technique), goal of sedation, or use of remifentanil as an adjunct may contribute to significant heterogeneity in the outcome parameters of mSaO2, duration of endoscopic examination, time to fall asleep, and satisfaction among endoscopic performers. Therefore, it remains controversial regarding the justification of combining the data from studies with different protocols to be analyzed as pooled results to reach a general conclusion. Further large-scale RCTs are warranted to verify our findings before their clinical application. Third, the lack of uniform criteria for documenting the degree of airway obstruction in the included studies rendered the comparison of this parameter impossible despite its potential effects on study outcomes. Fourth, the current study only focused on the comparison between propofol and dexmedetomidine in patients with OSA receiving DISE without investigating the effects of midazolam and these hypnotic agents, which require future RCTs for elucidation. Finally, although previous retrospective studies demonstrated that DISE may affect the decision-making process in adult patients diagnosed with OSA [42,43], the impacts of different anesthetics for DISE on long-term surgical outcomes were unavailable in the included trials to identify the optimal agent for the procedure. Further studies are warranted to address this issue.

## 5. Conclusions

The results of the current meta-analysis demonstrated that, compared with propofol, dexmedetomidine exhibited less suppression on pulmonary function as well as a higher degree of satisfaction among endoscopic performers for patients with obstructive sleep apnea receiving drug-induced sleep endoscopy, suggesting the justification of incorporating this anesthetic agent into the sedation protocol for this procedure to enhance patient safety. Nevertheless, taking into consideration its relatively low efficacy and high risk of failed sedation, further large-scale trials are warranted to shed light on the benefits of the combined use of this anesthetic with other agents in this clinical setting.

## Figures and Tables

**Table 1:** Characteristics of included studies.

Study	Age (year)	BMI (kg/m^2^)	AHI	Number	Goal of Sedation	Intervention	Comparison	Sedation Technique
Cho 2015 [24]	Pro = 41.7 ± 12.8 Dex = 40.8 ± 11.8	Pro = 25.4 ± 3.7 Dex = 25.9 + 3.5	Pro = 40.1 ± 26.0 Dex = 48.0 ± 20.1	42	OAA/S score of 3–5 or BIS of 55–75	Pro/Remi	Dex/Remi	Target-controlled infusion
Elkalla 2019 [23]	Pro = 38.6 ± 8.3 Dex = 39.4 ± 7.7	Pro = 27.3 ± 2 Dex = 26.9 ± 2.1	Pro = 32.2 ± 10.8 Dex = 30.7 ± 12	40	RSS ≥ 3	Pro	Dex	Infusion
Padiyara 2020 [22]	Pro = 40.7 ± 11.2 Dex = 40.6 ± 12.9	Pro = 30.1 ± 4.0 Dex = 29.2 ± 3.3	Pro = 55.9 ± 25.0 Dex = 48.6 ± 28.0	60	Beginning of snoring and BIS = 70	Pro	Dex	Infusion
Zhao 2018 [20]	Pro = 43.2 ± 6.6 Dex = 42.5 ± 6.0	Pro = 28.9 ± 3.1 Dex = 28.0 ± 3.5	Pro = 54.3 ± 20.4 Dex = 56.3 ± 21.5	88	Disappearance of eyelash reflex	Pro	Dex	Bolus and infusion
Kuyrukluyıldız 2015 [21]	Pro = 43.3 ± 10.6 Dex = 47.4 ± 11.6	Pro = 28.9 ± 3.9 Dex = 29.5 ± 4.1	NA	40	BIS < 75 and RSS = 4	Pro	Dex	Infusion

Pro: propofol; Dex: dexmedetomidine; AHI: apnea-hypoxia index; Remi: remifentanil; OAA/S: Observer’s Assessment of Alertness/Sedation Scale; BIS: bispectral index; RSS: Ramsey Sedation Scores; NA: not available.

## Supplementary Materials

The following are available online at https://www.mdpi.com/article/10.3390/jcm10081585/s1, Table S1: Literature Search for Conducting Systematic Review, Table S2: Definition of bradycardia and hypotension in each trial, Table S3: Risk of bias judged by authors.
