# Supplementary material for: The Use of Propofol versus Dexmedetomidine for Patients Receiving Drug-Induced Sleep Endoscopy: A Meta-Analysis of Randomized Controlled Trials
Source: J Clin Med. 2021 Apr 9;10(8):1585. doi: 10.3390/jcm10081585 (PMC8070183; doi:10.3390/jcm10081585)
Supplement: Supplementary file 1 [file jcm-10-01585-s001.pdf]

Journal of
Clinical Medicine

Supplemental Table 1: Literature Search for Conducting Systematic Review (Documentation Form)

Question and PICOS

Question	A comparison of propofol versus dexmedetomidine for patients receiving drug-induced sleep endoscopy
Population	Patients receiving drug-induced sleep endoscopy
Intervention	Dexmedetomidine was used as the intervention
Comparison	Propofol was used as the control
Outcome	Sedation outcomes (e.g., risk of oxygen desaturation or hypotension)
Study Type	Randomized Controlled Trial

Search strategies for Embase

	Item	Search Syntax
P	1	("DISE" OR "drug-induced sleep endoscopy" OR "sleep endoscopy"):ti,ab,kw,de
I	2	("Dexmedetomidine" OR "precedex" OR "Dexdor"):ti,ab,kw,de
C	3	("Propofol" OR "Diprivan" OR " 2, 6-diisopropylphenol"):ti,ab,kw,de
	4	#1 AND #2 AND #3 AND [embase]/lim
S	5	#4 AND ('crossover procedure':de OR 'double-blind procedure':de OR 'randomized controlled trial':de OR 'single-blind procedure':de OR (random* OR factorial* OR crossover* OR cross NEXT/1 over* OR placebo* OR doubl* NEAR/1 blind* OR singl* NEAR/1 blind* OR assign* OR allocat* OR volunteer*):de,ab,ti)

Supplemental table 2. Definition of bradycardia and hypotension in each trial

Study	Bradycardia	Hypotension
Cho 2015	HR <45 beats/min	MBP <60 mmHg
Kuyrukluyıldız 2015	HR <50 beats/minute for 15 seconds	MBP <70% baseline value for 60 seconds
Padiyara 2020	HR <50 bpm for 15 seconds	MBP <70% baseline value
Zhao 2018	HR <50 bpm	MBP ≤70% baseline value
Elkalla 2019	HR <50 beats/min	MBP <80% of baseline value

HR: heart rate; MBP: mean blood pressure

Supplemental table 3. Risk of bias judged by authors

Study: Cho 2015

Bias	Authors' Judgement	Support for Judgement
Random sequence generation	Low risk	Sixty-six patients were randomly assigned to one of the three study groups based on a computer-generated random table.
Allocation concealment	Low risk	The otorhinolaryngologist, patients, and anesthesiologists were unaware of the group identities.
Blinding of participants and personnel	High risk	No specific statement
Blinding of outcome assessment	High risk	No specific statement
Incomplete outcome data	Low risk	Among the 66 patients initially included in the study, two Dex-R group patients were eliminated because they refused DISE.
Selective reporting	Low risk	This trial was registered at clinicaltrial.gov (Ref NCT01895348). Information regarding primary and secondary outcomes were available in this study.
Other bias	Low risk	The authors have no funding, financial relationship, or conflicts of interest to disclose.

Study: Elkalla 2019

Bias	Authors' Judgement	Support for Judgement
Random sequence generation	Low risk	The randomization was done using computer-generated random numbers.
Allocation concealment	Low risk	The randomization was concealed in sealed opaque envelopes. A blinded nurse, who did not participate in the study or data collection, read the number contained in the envelope and made group assignments.
Blinding of participants and personnel	Low risk	The study drugs were labeled as infusion A, B, and C respectively, and administered by an anesthesiologist not participating in data collection. As the colors and the rate of infusion of the study solutions were not identical, he covered all syringes and infusion sets as well as the screen of the syringe pumps by aluminium foil paper to assure blindness of the study.
Blinding of outcome assessment	Low risk	No specific statement
Incomplete outcome data	Low risk	The data for all of these patients were analyzed
Selective reporting	Low risk	The study was registered in Pan African Clinical Trials Registry (PACTR201706002232945). Information regarding primary and secondary outcomes were available in this study.
Other bias	Low risk	The authors have no funding, financial relationship, or conflicts of interest to disclose.

Study: Kuyrukluyıldız 2015

Bias	Authors' Judgement	Support for Judgement
Random sequence generation	Uncertain risk	No specific statement
Allocation concealment	High risk	No specific statement
Blinding of participants and personnel	High risk	No specific statement
Blinding of outcome assessment	High risk	No specific statement
Incomplete outcome data	Low risk	The data for all of these patients were analyzed
Selective reporting	Low risk	Although the registered information about trial was not mentioned, information regarding primary and secondary outcomes were available in this study.
Other bias	Low risk	The authors have no funding, financial relationship, or conflicts of interest to disclose.

Study: Padiyara 2020

Bias	Authors' Judgement	Support for Judgement
Random sequence generation	Low risk	All the patients were randomized to either Group P (propofol; n = 30) or Group D (dexmedetomidine; n = 30) using the computer-generated, random-number-based, sealed-envelope method.
Allocation concealment	Low risk	All the patients were randomized to either Group P (propofol; n = 30) or Group D (dexmedetomidine; n = 30) using the computer-generated, random-number-based, sealed-envelope method.
Blinding of participants and personnel	Low risk	The observations of VOTE classification were made by the investigator (S.B.), who was blinded to the intervention. The drugs were given by the anesthetist not involved in the anesthesia protocol.
Blinding of outcome assessment	Low risk	The recordings of time to achieve sedation, hemodynamic response, and desaturations were made by the blinded observer not involved in the drug delivery.
Incomplete outcome data	Low risk	There were no voluntary dropouts in this study.
Selective reporting	Uncertain risk	The registered information about trial was not mentioned. Only some information regarding primary and secondary outcomes were available in this study.
Other bias	Low risk	The authors have no funding, financial relationship, or conflicts of interest to disclose.

Study: Zhao 2018

Bias	Authors' Judgement	Support for Judgement
Random sequence generation	Low risk	All patients were assigned by dint of a computer-generated random number table into one of two groups (n = 44): group D and group P.
Allocation concealment	Uncertain risk	No specific statement
Blinding of participants and personnel	Low risk	Drug induced sleep was carried out by one experienced anesthetist, and the otolaryngologist was blinded to which kind of drug performed endoscopy.
Blinding of outcome assessment	Low risk	All related data were collected by another anaesthetist not directly involved in anaesthesia for the patient.
Incomplete outcome data	Low risk	A total of 88 patients enrolled successfully completed DISE with propofol and dexmedetomidine in this study, and the data for all of these patients were analyzed.
Selective reporting	Low risk	This trial is registered at the Chinese Clinical Trial Registry (ChiCTR-IOR-17010423). The information regarding primary and secondary outcomes were available in this study.
Other bias	Low risk	The authors have no funding, financial relationship, or conflicts of interest to disclose.
